# Supplementary material for: The Role of Incentives in Deciding to Receive the Available COVID-19 Vaccine in Israel
Source: Vaccines (Basel). 2022 Jan 4;10(1):77. doi: 10.3390/vaccines10010077 (PMC8778303; doi:10.3390/vaccines10010077)
Supplement: Supplementary file 1 [file vaccines-10-00077-s001.zip › vaccines-1517712-supplementary.pdf]

Table S1. Items for assessing measures of HBM and incentives.

Model	Measures	Items
**HBM**	Perceived susceptibility	I believe that if I do not get vaccinated, the likelihood of me getting infected with corona will increase
		I believe that if I do not get vaccinated, the likelihood of my family and relatives getting infected in Corona will increase
	*Perceived severity	Even if I will get infected with COVID-19 I do not think it will cause me significant suffering or complications
		Even if I get infected with COVID-19, the likelihood of recovering from the disease is very high
	Perceived Benefits	I believe that COVID-19 vaccine will have high efficacy in preventing significant suffering and complications of the disease
		I believe that if I get vaccinated against COVID-19 the risk of getting infected with the disease or infecting others will decrease
	Perceived barriers	Getting vaccinated is expensive, requires time and effort
	Cues to action	The chances of me getting vaccinated against COVID-19 will increase if opinion leaders on social media express support for the benefit of the vaccine
		The chances of me getting vaccinated against COVID-19 will increase if friends and family express support for the benefit of the vaccine
		The chances of me getting vaccinated against COVID-19 will increase if official guidelines from the Ministry of Health are published
		The chances of me getting vaccinated against COVID-19 will increase if my GP recommends me
		If my workplace takes care of vaccinating the workers against COVID-19, I will vaccinate
	Health motivation	I exercise as recommended for my age
		I make sure to eat a healthy and varied diet
**Incentives**	Availability	If the vaccine is accessible and available
	Monetary reward	If I receive a monetary reward for getting vaccinated
	Green pass	If I receive a "green pass" that will allow various reliefs (entry to places of entertainment etc.)
	Monetary penalty	If the government cuts my social security benefits or imposes another fine if I do not get vaccinated

a Cronbach indicates the internal consistency: **HBM** α=0.796
Items Response scale: 1-6 agreement
* Negative items were reverse scored.

Table S2: Univariate analyses between HBM, incentives variables and willingness to get vaccinated against COVID-19

	DO not-intend to get vaccinated (n= 86)		Intend to get vaccinated (n= 375)		t-test	P value (two-tail)
**Variables**	Mean (SD)		Mean (SD)			
**HBM variables**						
Perceived Susceptibility	2.84	(1.39)	5.04	(1.21)	-14.67	<.001
Perceived Severity	2.68	(1.42)	2.98	(1.24)	-1.96	.051
Perceived Benefits	3.40	(1.32)	5.38	(.75)	-13.39	<.001
Perceived Barriers	3.22	(1.76)	3.04	(1.67)	.86	.39
Cues to action	2.10	(1.12)	4.21	(1.15)	-15.41	<.001
Health motivation	4.21	(1.45)	4.00	(1.39)	1.23	.220
**Incentives variables**						
Availability	2.09	(1.14)	5.31	(1.21)	-22.50	<.001
Monetary reward	1.69	(1.18)	3.03	(2.05)	-5.84	<.001
Green pass	2.88	(1.88)	4.91	(1.59)	-9.30	<.001
Monetary penalty	2.81	(1.90)	3.95	(1.94)	-4.93	<.001

Note: COVID-19 vaccination intention measured by the item: “I want to get vaccinated against the COVID-19 virus now that a vaccine is available”, on a 1-6 agreement scale
HBM and incentives Items Response scale: 1-6 agreement

Table S3: Univariate analyses between HBM, incentives variables and the sense of urgency to receive COVID-19 vaccine

	Immediately (n= 279)		Within 3 months (n= 73)		Within a year (n= 75)		F-test	P value
**Variables**	Mean	(SD)	Mean	(SD)	Mean	(SD)		
**HBM variables**								
Perceived Susceptibility	5.23	(1.11)	4.27	(1.35)	3.86	(1.46)	46.19	<.001
Perceived Severity	3.06	(1.28)	2.80	(1.15)	2.80	(1.28)	2.08	.127
Perceived Benefits	5.49	(.72)	4.97	(.76)	4.32	(1.18)	63.58	<.001
Perceived Barriers	2.96	(1.68)	3.32	(1.65)	3.30	(1.70)	2.13	.12
Cues to action	4.41	(.99)	3.72	(1.18)	2.80	(1.32)	67.20	<.001
Health motivation	3.90	(1.41)	4.23	(1.39)	4.11	(1.31)	1.84	.161
**Incentives variables**								
Availability	5.64	(.88)	4.61	(1.28)	2.84	(1.42)	209.45	<.001
Monetary reward	3.07	(2.11)	2.76	(1.86)	2.47	(1.69)	2.92	(.06)
Green pass	5.11	(1.52)	4.48	(1.62)	3.58	(1.80)	28.39	<.001
Monetary penalty	4.13	(1.97)	3.35	(1.78)	3.44	(1.78)	7.15	.001

Note: the sense of urgency to receive COVID-19 vaccine measured by the item: "now as the vaccine is available, how soon will you get vaccinated? Immediately, within 3 months or within a year?".
HBM and incentives Items Response scale: 1-6 agreement
